# Transcriptomic Analysis of Wheat Seedling Responses to the Systemic Acquired Resistance Inducer N-Hydroxypipecolic Acid

**DOI:** 10.3389/fmicb.2021.621336

**Published:** 2021-02-11

**Authors:** Eric T. Zhang, Hao Zhang, Weihua Tang

**Affiliations:** ^1^National Key Laboratory of Plant Molecular Genetics, Center for Excellence in Molecular Plant Sciences, Institute of Plant Physiology and Ecology, Chinese Academy of Sciences, Shanghai, China; ^2^Shanghai High School International Division, Shanghai, China; ^3^Department of Biology, University of Texas at San Antonio, San Antonio, TX, United States

**Keywords:** pathogen, fungal infection, systemic acquired resistance, Fusarium head blight (FHB), wheat, immune receptor

## Abstract

The fungal pathogen *Fusarium graminearum* can cause destructive diseases on wheat, such as Fusarium head blight and Fusarium crown rot. However, a solution is still unavailable. Recently, N-hydroxypipecolic acid (NHP) was identified as a potent signaling molecule that is capable of inducing systemic acquired resistance to bacterial, oomycete, and fungal infection in several plant species. However, it is not clear whether NHP works in wheat to resist *F. graminearum* infection or how NHP affects wheat gene expression. In this report, we showed that pretreatment with NHP moderately increased wheat seedling resistance to *F. graminearum*. Using RNA sequencing, we found that 17% of wheat-expressed genes were significantly affected by NHP treatment. The genes encoding nucleotide-binding leucine-rich repeat immune receptors were significantly overrepresented in the group of genes upregulated by NHP treatment, while the genes encoding receptor-like kinases were not. Our results suggested that NHP treatment sensitizes a subset of the immune surveillance system in wheat seedlings, thereby facilitating wheat defense against *F. graminearum* infection.

## Introduction

Wheat (*Triticum aestivum*) is one of the main sources of calories for humans (Food and Agriculture Organization of the United Nations, 2018). The devastating disease, Fusarium head blight (FHB), is threatening global wheat production and food safety (Goswami and Kistler, 2004; Ma et al., 2013). The filamentous fungus *Fusarium graminearum* is a causative pathogen of FHB in many countries, including the United States, China, and Canada. However, a solution to this serious problem is still unavailable (Bai and Shaner, 2004; Figueroa et al., 2018). Therefore, it is worthy to explore more effective means to control the disease.

Systemic acquired resistance (SAR) is a defense mechanism in plants that can be triggered by microbial attacks in part of the plant and provides the whole plant with enhanced resistance to later pathogen infection within several days (Shah and Zeier, 2013). Recently, N-hydroxypipecolic acid (NHP) was identified as a signaling molecule that activates SAR in *Arabidopsis* (Hartmann et al., 2018) and tomato (Holmes et al., 2019). Pretreated with exogenous NHP, these dicotyledonous plants increased resistance to both bacterial and oomycete pathogens (Hartmann et al., 2018). In addition, NHP accumulation was detected in *Magnaporthe oryzae*-inoculated barley and *Brachypodium distachyon*, and with exogenous NHP pretreatment, these monocotyledonous plants enhanced resistance to the fungal pathogen *M. oryzae* (Schnake et al., 2020). These results indicated a potential of using NHP to improve crop resistance to pathogens. However, it has not been reported whether NHP pretreatment also provides resistance to *F. graminearum* in wheat.

During the infection of *Arabidopsis*, pipecolic acid (Pip) is synthesized at the infection site from lysine by L-lysine alpha-aminotransferase (ALD1) and a reductase [SAR DEFICIENT 4 (SARD4)] (Ding et al., 2016). Then, flavin-dependent monooxygenase 1 (FMO1) catalyzed the one-step biochemical conversion of Pip to NHP in *Arabidopsis* (Hartmann et al., 2018). Thereafter, NHP can be transported through the phloem and triggers defense responses in distant parts of the plant. The distant cell responses include increasing the expression of *ALD1* by the WRKY33 transcription factor (Wang et al., 2018; Sun et al., 2020), which further leads to NHP biosynthesis. In *Arabidopsis*, transcriptomic response after 24-h treatment with exogenous Pip includes upregulation of a whole battery of plant immune-related and SAR-relevant genes, which underlies Pip-triggered resistance (Hartmann et al., 2018). Furthermore, Pip causes *Arabidopsis* transcriptomic response through NHP (Hartmann et al., 2018). However, a transcriptomic response to NHP treatment has not been reported. It also remains unclear whether NHP can induce a similar transcriptomic response in wheat.

In the present study, we reported that NHP pretreatment facilitates wheat seedling defense against *F. graminearum* and further investigated the global transcriptional changes in wheat upon NHP pretreatment, which will provide clues concerning the mechanism of action of NHP.

## Materials and Methods

### Wheat Inoculation Assay

Wheat (*T. aestivum*) cultivars Zhongyuan 98-68, Bobwhite, and Wangshuibai were used for inoculation. Wheat inoculation by *F. graminearum* strain PH-1 was performed as previously described (Jia et al., 2017) with minor modifications. Wheat seeds were imbibed overnight and placed in 24-well cell culture plates for germination. After 1 day of germination, when the shoots of seedlings were about 1 cm long, the top 1–2 mm of the coleoptiles were removed, and 1 μl NHP solution or water (control) was added to the wounded site. One day after NHP application, the top of coleoptiles of 3-day-old seedlings was cut again, and 1 μl of *F. graminearum* spore suspension (2 × 10^6^ spores per ml) was added to the wounded site. One day after spore inoculation (DAI), faint brown lesions at the top of the coleoptiles became visible, and at 3 DAI, the lesions became dark brown. The dark lesions extended down from the top of the coleoptiles, and at 7 DAI, the seedlings were photographed for lesion measurement. The lesion length was measured using ImageJ (Rasband, 1997–2018). For fungal biomass determination, aerial tissues of infected seedlings were harvested at 4 DAI for genomic DNA extraction using a fungal genomic DNA fast extraction kit (Sangon Biotech). SYBR Green detection system was used on an iCycler (Bio-Rad) to perform quantitative real-time PCR experiments using genomic DNA as templates. Primers of *F. graminearum* elongation factor 1α (Yang et al., 2010b) and a wheat reference gene (Yang et al., 2010a) are listed in Supplementary Table 1.

### RNA Sequencing

Wheat (*T. aestivum*) cultivar Zhongyuan 98-68 was planted in 24-well cell culture plates (one seed per well) in a 25°C incubator. After 3 days, three plates of seedlings were treated with 1 μl of 1 mM NHP per seedling. The other three were treated with water and used as the control group. The coleoptiles were collected at 1 day after treatment. Each plate served as a biological replicate for its treatment group. Total RNA was extracted using an EasyPure Plant RNA kit (Transgen Biotech, Beijing, China) according to the manufacturer’s protocol. RNA quality was evaluated using a Bioanalyzer 2100 (Agilent Technology, Santa Clara, CA, United States). The RNA sequencing (RNA-seq) was performed at Sinotech Genomics (Shenzhen, China). The libraries for sequencing were prepared using a SureSelect Strand-Specific RNA Component kit (Agilent Technology, Santa Clara, CA, United States). RNA-seq was performed using an Illumina NovaSeq 6000 machine (Illumina, San Diego, CA, United States) to obtain 150 nt paired-end reads.

### RNA Sequencing Data Analysis

Sequencing reads were preprocessed using Trim Galore (Felix, 2019) to trim the adapter sequences and then mapped to the genome sequence assembly of *T. aestivum*, IWGSC 47,^1^ using HISAT2 (Kim et al., 2015) with strand-specific information (–rna-strandness RF) and other settings at default. Only the uniquely mapped reads were used to create a count matrix using featureCounts (Liao et al., 2014). Differential gene expression analysis was performed using DESeq2 (Love et al., 2014). To generate more accurate log_2_ fold change (LFC) estimates, the shrinkage of the LFC was conducted using the apeglm method within the lfcShrink function (Zhu et al., 2019). The Benjamini and Hochberg false discovery rate (FDR) procedure was used for multiple hypothesis testing corrections (Benjamini and Hochberg, 1995). Genes with an FDR-adjusted *p* value <0.05 and an LFC of more than 1 or less than −1 were considered to be differentially expressed. Gene Ontology (GO) enrichment analysis was performed with the clusterProfiler package of R and the enrichment criteria including a corrected *p* value <0.05 (Yu et al., 2012). Heatmaps of specific genes were generated using the pheatmap package of R (Kolde, 2012).

### Reverse Transcription Quantitative PCR

Total RNA was extracted from 3-day-old wheat coleoptiles of the NHP- or mock-treated groups using the method stated in section “RNA Sequencing.” cDNA was prepared using a Transcript One-Step gDNA Removal and cDNA Synthesis SuperMix (Transgen Biotech, Beijing, China). Nine genes were selected randomly for validation of RNA-seq results. The SYBR Green detection system was used on an iCycler instrument (Bio-Rad, Hercules, CA, United States) to perform the quantitative real-time PCR reactions. *TraesCS7A02G276400*, which encodes a chlorophyll a-b binding protein, was used as the reference. The primers for these 10 genes are listed in Supplementary Table 1.

### Toxicity Assessment and Microscopic Analysis

To briefly assess NHP toxicity on *F. graminearum*, an aliquot of 100 μl *F. graminearum* spore suspension was added into 1.9-ml fresh liquid media (mung bean broth; Jia et al., 2017) supplemented with NHP, followed by 25°C 150 rpm incubation for 2 days. The spores of each culture were then collected and counted using a hemocytometer. The spore viability was assessed by propidium iodide staining [1.5 μM propidium iodide in 0.067 M phosphate buffer (pH 6.8)]. Then, the fungal samples were imaged on an Olympus BX51 microscope with a red fluorescent protein (RFP) filter set.

To briefly assess NHP effects on wheat cells, the coleoptiles of wheat seedlings at 24 h after water or NHP treatment were peeled and imaged on an Olympus BX51 microscope. Callose visualization was performed according to a report (Luna et al., 2011) with minor modifications. Detached coleoptiles (approximately 1 cm long from the top edge) from water- or NHP-treated seedlings were incubated for 24 h in 95% to 100% ethanol, were washed in 0.067 M phosphate buffer (pH 9), and were stained with 0.02% (w/v) aniline blue in 0.067 M phosphate buffer (pH 9) for 1 h prior to microscopic analysis. Epifluorescence pictures with UV filter were obtained at fixed setting and analyzed with ImageJ (Rasband, 1997–2018). Integrated intensities of callose signals were measured after background subtraction (setting rolling ball radius 30). The experiment was repeated twice. For each experiment, 10 individual seedlings per sample and three images per coleoptile of seedling were measured. Images at similar positions of coleoptiles were captured. Average intensities relative to water-treated sample were charted, and two-tailed Student’s *t*-test was performed for statistical analysis.

## Results and Discussion

### N-Hydroxypipecolic Acid Pretreatment Alleviates Disease Symptoms Caused by *F. graminearum*

**FIGURE 1:**
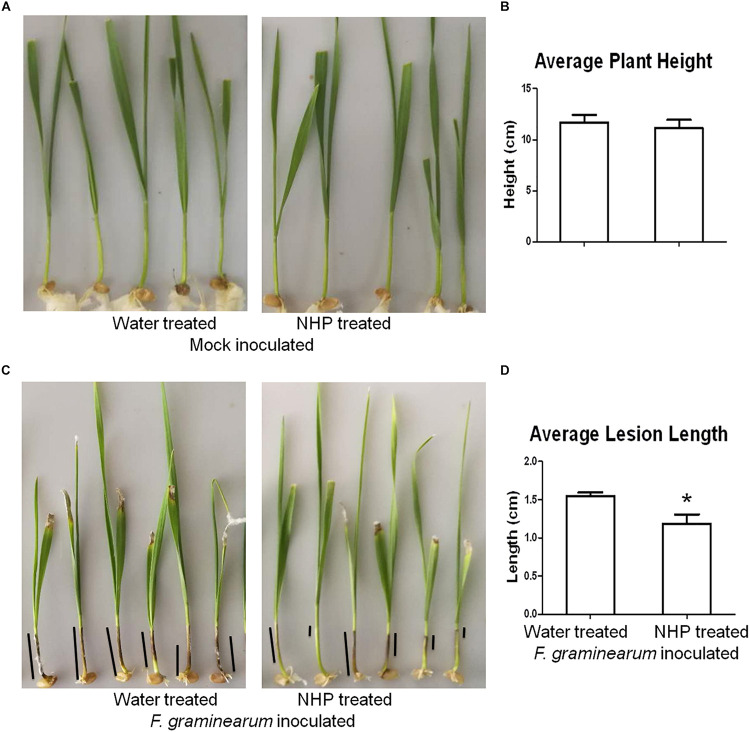
Representative images and measurements of wheat seedlings treated with water or N-hydroxypipecolic acid (NHP). **(A,B)** Mock-inoculated seedlings. **(C,D)** *F. graminearum*-inoculated seedlings. Black lines show examples of lesions on coleoptiles; *n* = 3 independent experiments. Error bars indicate the SE. An asterisk indicates a significant difference compared with the control (*p* < 0.05; Student’s *t*-test).

To investigate whether NHP pretreatment can enhance wheat resistance to *F. graminearum*, we used a coleoptile inoculation assay (Zhang et al., 2012). As shown in Figures 1A,B, applying 1 nmol per seedling NHP on the coleoptiles of 2-day-old seedlings of wheat cultivar Zhongyuan 98-68 did not affect plant growth significantly compared with that of the water-treated group. The inoculation of *F. graminearum* spores on the coleoptiles of 3-day-old seedlings produced dark brown lesions, which were approximately 1.5 cm long when measured at 7 DAI (Figures 1C,D). The wheat seedlings pretreated with 1 nmol NHP 1 day before *F. graminearum* spore inoculation developed lesions with similar appearance, but the lesions were approximately 1.2 cm long, which was 20% shorter than those without NHP treatment (Figures 1C,D). Pretreatment with 1 nmol per seedling NHP on two other wheat cultivars, Bobwhite and Wangshuibai, also caused 15–25% reduction on lesion lengths (Supplementary Figures 1, 2). The biomass of *F. graminearum* was approximately 18% lower in infected seedlings pretreated with NHP than that in those without NHP treatment (Supplementary Figure 1). In addition, concentration up to 160 μM NHP did not affect spore production by *F. graminearum* (Supplementary Figure 3). Propidium iodide staining of *F. graminearum* spores incubated with or without NHP showed similar patterns of fluorescent signals on cell walls, but not in cytoplasm, indicating that spores were viable (Supplementary Figure 4). These results indicate that the increased resistance to *F. graminearum* is unlikely to be achieved through inhibition of fungal growth, rather through enhancement of wheat defense.

**FIGURE 2:**
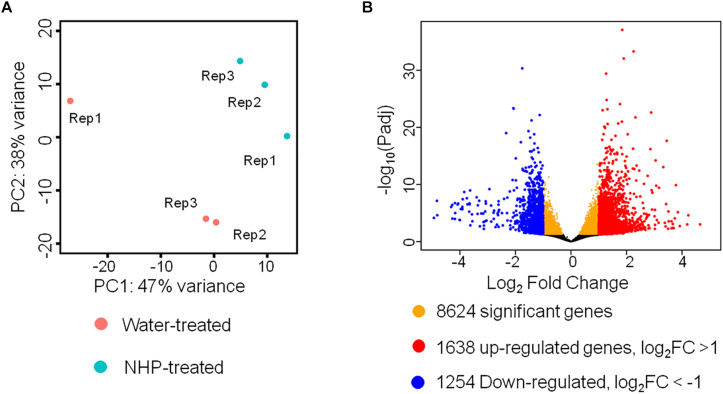
RNA sequencing results of wheat seedlings treated with water or N-hydroxypipecolic acid (NHP). **(A)** Principal component analysis (PCA) analysis of the transcriptomes of NHP-treated and water-treated wheat seedlings. **(B)** Volcano plot showing the distribution of differentially expressed genes. FC, fold change of NHP-treated sample with respect to mock-treated sample. False discovery rate (FDR) <0.05.

With this magnitude of resistance enhancement in wheat (i.e., approximately 20% reduction of disease severity), NHP treatment could be one of the methods used to jointly control *F. graminearum*-caused diseases because a single protection solution, such as a completely resistant cultivar or a fungicide that is effective under all conditions, is unavailable. On the other hand, NHP pretreatment in *Arabidopsis* and tobacco inhibited 90% of the *in planta* growth of the bacterial pathogen *Pseudomonas syringae* (Schnake et al., 2020). This implies a difference between NHP actions in wheat and in *Arabidopsis*/tobacco.

### N-Hydroxypipecolic Acid Can Cause Profound Changes of Wheat Transcriptome

To understand the underlying mechanisms of NHP function, we explored the wheat global gene expression changes caused by NHP pretreatment. We performed RNA-seq to compare the transcriptomes of wheat seedlings with or without NHP treatment. Three biological replicates for each sample (NHP-treated and water-treated) were collected and sequenced using Illumina NovaSeq 6000.

We detected 50,696 expressed unigenes, which account for 42% of 120,744 annotated genes in wheat genome. Principal component analysis (PCA) was applied to visualize the overall transcriptomic similarities between wheat samples at 1 day after treatment with NHP and those at 1 day after treatment with water. As shown in Figure 2A, the transcriptomes of three NHP-treated samples were clustered close to each other and were separate from those of the water-treated samples, indicating high reproducibility within the NHP-treated samples and distinctive global expression between NHP-treated and water-treated wheat samples. This result indicated that NHP treatment profoundly affects wheat gene expression. Using a Benjamini–Hochberg adjusted *p* value of 0.05 as the statistical significance threshold, we identified 8,624 differentially expressed genes (DEGs) between NHP-treated and water-treated samples, i.e., the expression levels of 17% of 50,696 expressed genes changed significantly upon NHP treatment (Figure 2B). Based on the threshold of a twofold (up- or down-) change in expression level and an adjusted *p* value <0.05, we identified 2,892 genes that showed a significant difference in expression between the NHP-treated and water-treated samples, among which 1,638 genes were upregulated and 1,254 were downregulated in NHP-treated wheat samples relative to the water-treated controls (Figure 2B).

**FIGURE 3:**
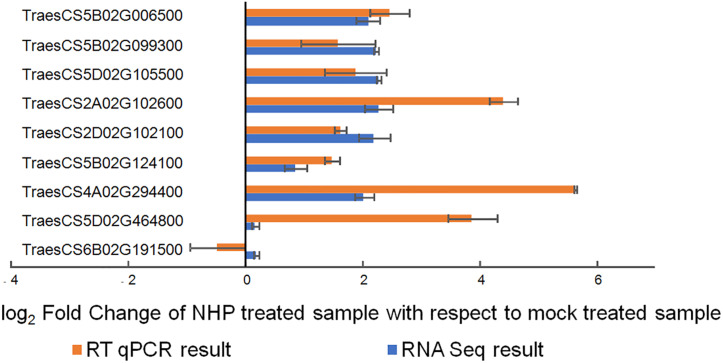
Quantitative real-time reverse transcription quantitative PCR (qRT-PCR) verification of the expression of nine wheat genes upon N-hydroxypipecolic acid (NHP) treatment. Error bars indicate the SE, *n* = 3.

We then performed reverse transcription quantitative PCR on nine randomly selected genes using independent RNA samples as templates. Seven genes (78%) showed results that were highly consistent with the RNA-seq results (Figure 3), indicating the reliability of the transcriptomic results.

### Putative Intracellular Immune Receptors and Associated Genes Are Among the Top N-Hydroxypipecolic Acid-Upregulated Genes

**FIGURE 4:**
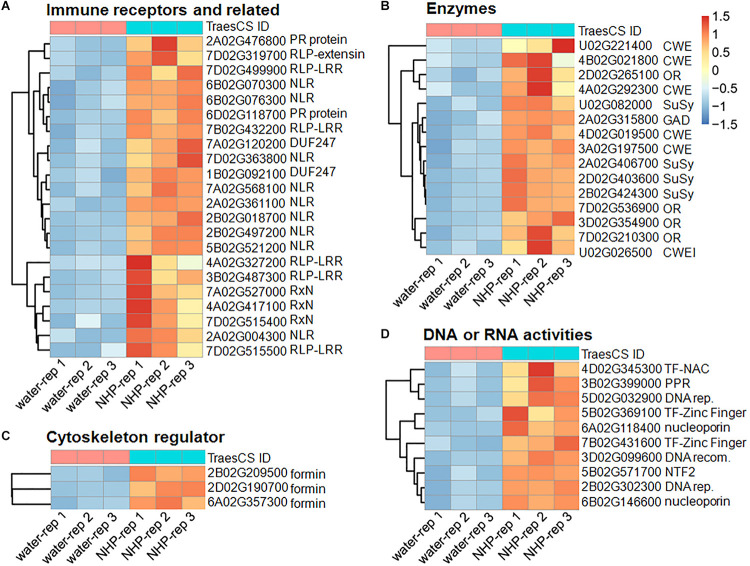
Heatmap presentation of the top 50 upregulated genes in response to N-hydroxypipecolic acid (NHP) treatment. Normalized expression data were presented. **(A)** Genes in group 1 and 2. **(B)** Genes in group 3, 4 and 5. **(C)** Genes in group 6. **(D)** Genes in group 7 and 8.

To find clues of NHP action mechanisms, we first focused on those genes that were upregulated upon NHP treatment by assigning putative functions of the top 50 upregulated genes (Figure 4). The top 50 upregulated genes, whose expression increased at least sixfold in 1 day after NHP treatment, could be assigned to eight gene groups based on the annotated functions of their encoded protein (Supplementary Table 2).

The first group of upregulated genes comprised nine genes encoding nucleotide-binding domain and leucine-rich repeat (NLR) proteins (Figure 4A). NLR family members are known as plant intracellular immune receptors that monitor pathogen effectors or the changes caused by effectors, and trigger host plant immunity, including hypersensitive responses (Adachi et al., 2019). The wheat genome contains 2,151 NLR genes, which account for almost 1.8% of the predicted genes in the genome (Bouktila et al., 2014; Sekhwal et al., 2015; Andersen et al., 2020), among which 648 were detected as expressed in our RNA-seq (accounting for approximately 1.3% of 50,696 expressed genes). Notably, 18% of the top 50 upregulated genes and 5.8% (95) of the total 1,638 upregulated genes encode NLRs. Among the 95 NLR proteins encoded by NHP-upregulated genes, 79 contained potato virus X resistance protein N-terminal coiled-coil (CC) domain (InterPro IPR041118 domain), 72 contained LRR domains, two contained protein kinase domain (InterPro IPR000719 domain), two contained winged helix-turn-helix DNA-binding motif (InterPro IPR036388 domain) (Gajiwala and Burley, 2000), and one contained Armadillo-type fold (InterPro IPR016024 domain) (Groves and Barford, 1999). None of those upregulated NLRs contained resistance to powdery mildew 8 (RPW8) domain (Wang et al., 2009), Toll/interleukin-1 receptor homology (TIR) domain, WRKY domain, or Myb domain. By contrast, none of the top 50 downregulated genes belonged to the NLR family, and only two (0.16%) of the 1,254 downregulated genes belong to the NLR family. Their significant enrichment among the upregulated genes (particularly in the top 50 upregulated group) and their scarcity among the downregulated group showed that NLR genes mainly increase expression in response to NHP.

The second group in the top 50 are components of or associated with plant immune system, including five genes encoding leucine-rich repeats containing proteins (RLPs), two genes encoding putative pathogenesis-related (PR) proteins, three genes encoding proteins containing potato virus X resistance protein N-terminal (RxN) domain, and two genes that encode proteins containing the domain of unknown function, DUF247 (Figure 4A). The RxN domain is also present in the N-termini of a major type of NLR proteins, termed coiled-coil domain (Hao et al., 2013). Six out of the nine NLRs in the first group of the top 50 contain the RxN domain. The DUF247 domain has been reported to be present in some NLR protein C-termini as an “integrated domain” implicated in facilitating NLR targeting pathogen effectors (Sarris et al., 2016).

Plant immune surveillance system mainly comprises two types of receptors, intracellular NLRs and cell-surface receptors. Along with receptor-like kinases (RLKs), RLP members constitute the cell-surface immune receptors (Zhou and Zhang, 2020). Given that the wheat genome encodes over 2,000 RLPs and RLKs, genes encoding cell-surface immune receptors are not enriched among the upregulated genes. It has been reported that tomato lines with surface-localized immune receptors I and I-3 were more effective at restricting *Fusarium* spread than those with NLR I-2 (van der Does et al., 2019). This might partially explain why NHP pretreatment only moderately increased wheat resistance to *F. graminearum*.

The PR proteins are not or barely detectable under healthy conditions; however, they accumulate at the protein level under pathological conditions and, therefore, are a part of the defense response, being components of the plant immunity (Sels et al., 2008). We particularly examined the expression of all 39 PR-like genes in wheat (Supplementary Table 3). The expression levels of most of them did not change significantly, and only two were upregulated significantly (Figure 4A), and one PR-5-like, one PR-4-like, and one PR-3-like genes were downregulated significantly.

The non-expresser of pathogenesis-related (NPR) proteins are receptors of the plant defense hormone salicylic acid (SA; Wang et al., 2020). Among the 12 expressed NPR-like genes, only one, *NPR4-A*, was significantly upregulated (with a fold change >2). The NPR1-like genes were not upregulated by NHP. In summary, except for the intracellular immune receptors, which were significantly enriched, other components of plant immune system, such as cell-surface receptors, were not significantly enriched in the top upregulated group, suggesting that NHP induces a subset of the plant immune system genes.

### N-Hydroxypipecolic Acid Treatment Also Increases the Expression of Cell Wall Fortification Enzymes

The third to fifth groups among the top 50 upregulated genes encoded various enzymes (Figure 4B), whose functions have been annotated in GO. The third group of genes encoded four diverse oxidoreductases, including a Laccase, a secreted peroxidase, a cupredoxin, and an axoglutarate/iron-dependent dioxygenase, which might be related to defense redox changes. Overall oxidoreductase activity GO terms were not enriched in the upregulated genes. Laccase and secreted peroxidase have been reported to function to cross-link cell wall components (Giardina and Sannia, 2015). It is possible that the upregulation of these genes is relevant to cell wall fortification.

The fourth group in the top 50 upregulated genes encoded four sucrose synthases, which catalyze sucrose synthesis and cleavage, serving as direct and reversible regulators of sucrose flux (Zheng et al., 2011). Among 17 sucrose synthase genes that were expressed in wheat seedlings, nine were upregulated. By contrast, none of sucrose synthase genes were downregulated. Furthermore, the top 30 GO terms enriched in the 1,638 upregulated genes (Supplementary Table 4) included sucrose metabolic process, sucrose synthase activity, and sucrose-phosphate synthase activity (involving 16 genes). Sucrose transport is central for the allocation of carbon resources in vascular plants. The upregulation of carbon resources allocation-related genes might be relevant to increased biosynthesis of cell wall components (suggested by the fifth group below).

**FIGURE 5:**
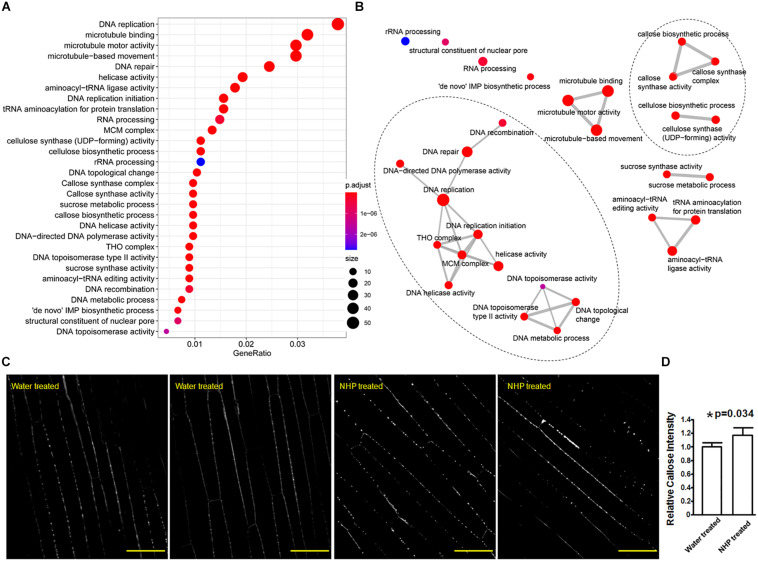
Gene Ontology (GO) terms that were overrepresented among the upregulated genes and callose deposition in wheat coleoptiles. **(A)** Dot plot presentation. **(B)** Map presentation. **(C)** Representative images of aniline blue-stained wheat coleoptiles. Scale bar = 50 μm. **(D)** Measurements of callose signals. *Significant difference detected by Student’s *t*-test.

The fifth group genes encoded cell wall-modification enzymes, including a callose synthase (also called β-1,3-glucan synthase), a wax ester synthase, three xylanases, and a xylanase inhibitor (Figure 4B). Furthermore, the top 30 GO terms significantly overrepresented in the 1,638 upregulated genes (Figure 5A and Supplementary Table 4) included five terms related to biosynthesis of the cell wall components callose and cellulose: 1,3-β-D-glucan synthase complex, 1,3-β-D-glucan synthase activity, (1–>3)-β-D-glucan biosynthetic process, cellulose synthase (UDP-forming) activity, and cellulose biosynthetic process (Figure 5B). There were 13 putative callose synthase genes and 15 cellulose synthase genes among the 1,638 upregulated genes. The function of these enzymes can be considered as defense-related cell wall fortification.

In particular, among all the 34 wheat genes encoding putative callose synthase that were expressed in our RNA-seq, only 10 of them showed no expressional change, 24 showed significantly higher expression in NHP-treated samples than in water-treated samples, with 11 out of these 24 showing a fold change less than two, while the other 13 showed a fold change ranging from 2 to 3.8 (Supplementary Table 5). So, this clearly showed the major increasing trend of callose synthase expression upon NHP treatment. Callose is a β-(1,3)-glucan polymer that can serve as a barrier to hinder pathogen hyphal extension (Luna et al., 2011). Using aniline blue staining, we also observed that callose signals discretely lined along cell walls of wheat coleoptiles with and without NHP treatment (Figure 5C). Assessed by the intensity of callose deposition, statistical analysis showed that coleoptiles pretreated with NHP accumulated approximately 16% more callose than those pretreated with water, as shown in Figure 5D, which is consistent with the overall NHP upregulation of callose synthase genes.

### N-Hydroxypipecolic Acid Treatment Results in Upregulation of Genes Associated With DNA Replication, Repair, and Related Activities

The sixth group encoded three formin-like proteins (Figure 4C). Thirty-seven formin-like proteins were expressed in our RNA-seq data, and genes encoding eight formin-like proteins are included in the 1,638 upregulated genes; none of these genes were downregulated. Formin functions as an actin cytoskeleton regulator, which might underlie cell growth. Related to this, microtubule motor activity, microtubule-based movement, and microtubule binding, as three microtubule cytoskeleton-related functions, were among the top 30 GO terms enriched in the 1,638 upregulated genes (Figure 5).

The last two groups are related to activities associated with DNA regulation (Figure 4D). The seventh group in the top 50 upregulated genes encoded three putative transcription factors, including two zinc-finger and one NAC domain-containing protein. The eighth group encoded six proteins with putative functions in DNA replication, recombination, and/or nucleocytoplasmic transport, including one similar to BRCA2 (breast cancer susceptibility gene 2), one similar to protein downstream neighbor of Son (DONSON), and two nuclear pore complex component nucleoporins. These putative transcription factors and nucleocytoplasmic transport-related proteins might function in preparing transcriptional reprogramming. Related to this, the top 30 GO terms that overrepresented among the upregulated genes (Supplementary Table 4) included 14 terms that are related to DNA replication and recombination, including DNA replication mini-chromosome maintenance (MCM) complex, DNA replication initiation, helicase activity, a suppressor of the Transcriptional defects of Hpr1 mutants by Overexpression (THO) complex, DNA topological change, DNA topoisomerase type II activity, DNA repair, DNA helicase activity, DNA-directed DNA polymerase activity, DNA metabolic process, DNA recombination, structural constituent of nuclear pore, and DNA topoisomerase activity (Figure 5). For example, the MCM complex is a hexameric protein complex required for the initiation and regulation of DNA replication (Lei and Tye, 2001). The THO complex is a nuclear complex that is required for transcription elongation through genes containing tandemly repeated DNA sequences (Luna et al., 2019). The putative functions of these groups imply chromosome reorganization and transcriptional reprogramming.

### N-Hydroxypipecolic Acid Treatment Results in Downregulation of Genes Related to Cell Redox Homeostasis

**FIGURE 6:**
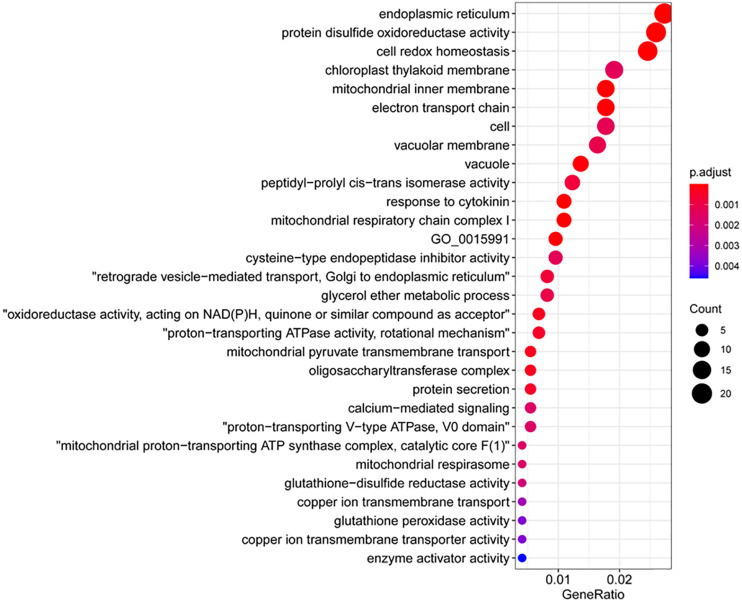
Gene Ontology (GO) terms that were overrepresented among the downregulated genes.

We then examined the significantly downregulated genes upon NHP treatment. The majority of the top 50 downregulated genes (>6.3-fold change) were hypothetical genes with unknown characterized or predicted functions, except for three genes that encode a Rhodanese-like domain-containing protein, a START-like domain-containing protein, and a zinc-finger containing protein (Supplementary Table 6). The GO terms enriched in the 1,254 downregulated genes belong to categories very different from those enriched in the upregulated genes. The top 30 GO terms that were overrepresented in the downregulated genes mainly comprised cell redox homeostasis and cellular response to oxidative stress (particularly related to protein disulfide oxidoreductase activity, glutathione-disulfide reductase activity, and glutathione peroxidase activity), protein modification (including peptidyl-prolyl cis-trans isomerase activity, oligo saccharyl transferase complex, cysteine-type endopeptidase inhibitor activity, cyclin-dependent protein serine/threonine kinase inhibitor activity, and enzyme activator activity) and calcium-mediated signaling (Figure 6). It seems that NHP pretreatment might cause cells to reduce their control of reduction–oxidation homeostasis.

### Wheat Transcriptomic Response to N-Hydroxypipecolic Acid Partially Overlaps With Systemic Acquired Resistance Response

As a mobile signal of SAR, NHP is transported from the local infection site to far away parts, where it further induces NHP production, which is a critical step in defense amplification (Shan and He, 2018). We examined the expression changes of putative NHP biosynthesis genes identified based on their homology to *Arabidopsis* enzymes ALD, SARD4, and FMO1 (Huang et al., 2020). In wheat, because of the large number of homologous family members, it is difficult to pinpoint the functional orthologs of these enzymes. Among 12 *ALD*-like genes and 22 *FMO1*-like genes, three *ALD*-like genes were significantly upregulated with a greater than 2-fold change and one *FMO1*-like gene was upregulated by 1.5-fold (Supplementary Table 3). These expression data suggested an increase of NHP production upon NHP treatment in wheat, which is consistent with the detection of NHP accumulation in two monocotyledonous plants (Schnake et al., 2020). NHP production upon pathogen infection was first identified in *Arabidopsis* (Hartmann et al., 2018) and then in tomato (Holmes et al., 2019). This result also supports the view that the positive feedback loop of NHP signal amplification is conserved in dicotyledonous and monocotyledonous plants.

**FIGURE 7:**
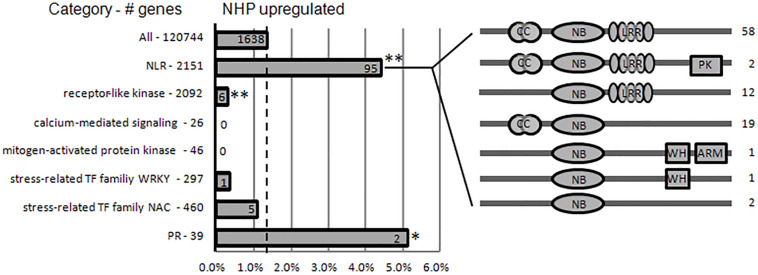
N-hydroxypipecolic acid (NHP) upregulated the expression of a subset of immune-related genes. Proportions of NHP-upregulated genes in families associated with immune perception, defense signaling, and immune executors. Broken line represents the percentage of NHP-upregulated genes in the whole wheat genome (120,744). The total number of genes in each category is provided on the left. The absolute numbers of NHP-upregulated genes in each category are provided on the bar. The domain structure of subclasses of upregulated nucleotide-binding domain and leucine-rich repeats (NLRs) are illustrated on the right. Asterisks indicate significant enrichment (or depletion) of gene categories in NHP-upregulated genes (**p < 0.005; *p < 0.05; chi-square test). NB-ARC, nucleotide-binding domain found in apoptotic protease activating factor 1, resistance genes, and *Caenorhabditis elegans* death-4 protein; LRR, leucine-rich repeats; CC, the potato virus X resistance protein N-terminal “coiled-coil” domain; WH, Winged helix DNA-binding domain; ARM, armadillo fold domain.

As an inducer of SAR, NHP is expected to induce SAR gene expression to enhance defense activation (Hartmann and Zeier, 2019). It has been reported that in *Arabidopsis*, elevation of Pip level activates transcriptional SAR responses (Hartmann et al., 2018). The gene families associated with the perception of pathogens (NLRs and receptor-like protein kinases), defense signaling (e.g., calcium and mitogen-activated protein kinase signaling), stress-related transcription factor families (WRKY and NAC), and “redox”-related categories were strongly enriched within the Pip-induced genes in *Arabidopsis*. Pip induces the expression of FMO1, which converts Pip to NHP; therefore, Pip treatment should cause similar responses as those to NHP treatment. We then compared our NHP-treated wheat data to Pip-treated *Arabidopsis* data. Among the gene families enriched within Pip-induced genes, the gene families associated with the intracellular perception of pathogens (NLRs) were strongly enriched within NHP-upregulated genes, while gene families associated with the cell-surface perception of pathogens (RLKs), defense signaling (calcium-mediated signaling and mitogen-activated protein kinases), and stress-related transcription factor families (WRKY and NAC) were depleted within NHP-upregulated genes (Figure 7 and Supplementary Table 2). In addition, the calcium-mediated signaling gene category and “redox”-related categories were even enriched among the NHP-downregulated genes (Supplementary Table 6).

Benzothiadiazole (BTH) can induce SAR in plants; five wheat chemical-induced genes *WCI-1* to *-5* were previously identified as induced by BTH (Gorlach et al., 1996). Noteworthy, three of them, which are one copy of *WCI-1* gene (TraesCS4D02G122600), one copy of *WCI-3* gene (TraesCS4D02G122600), and one copy of *WCI-4* gene (TraesCS2A02G034000), were significantly upregulated by NHP. *WCI-1* encodes a dirigent protein that putatively functions in modulating cell wall metabolism (Paniagua et al., 2017), *WCI-3* encodes a sulfur-rich/thionin-like protein, and *WCI-4* encodes a putative thiol protease, all of which are implicated in immunity functions.

In summary, our results show NHP-induced wheat transcriptomic responses partially overlap but are distinct from SAR responses. Incapability in activating the whole set SAR responses might help explain why NHP pretreatment only moderately increases wheat resistance to *F. graminearum*. Wheat seedlings responded to NHP treatment at the mRNA level by increasing the expression of many NLR intracellular immune receptors that monitor pathogen infection; of callose and cellulose synthases and putative lignin cross-linking enzymes that fortify cell walls; sucrose metabolic enzymes that allocate carbon resources; and a large proportion of DNA activity-associated proteins that imply DNA replication, repair, recombination, and/or transcriptional reprogramming. NHP treatment also reduced the expression of many protein modification enzymes, glutathione and disulfide pathway redox homeostasis-maintaining enzymes. Collectively, these changes might prepare wheat to defend against pathogens, including *F. graminearum*.

## Data Availability Statement

The data presented in this study can be found below: https://www.ncbi.nlm.nih.gov/geo/query/acc.cgi?acc=GSE160041.

## Author Contributions

EZ performed the “wet” experiments, including wheat inoculation assay, RT-PCR, genomic DNA extraction, and RNA extraction. HZ performed the RNA-seq data processing and analysis. WT conceived the project and analyzed the data. All authors wrote the manuscript.

## Conflict of Interest

The authors declare that the research was conducted in the absence of any commercial or financial relationships that could be construed as a potential conflict of interest.

## Footnotes

1 ftp://ftp.ensemblgenomes.org/pub/plants/release-47/fasta/triticum_aestivum/dna/

## Supplementary Material

Supplementary Figure 1 Representative images, lesion size measurements, and fungal abundance assessments of wheat cultivar Wangshuibai seedlings infected by *F. graminearum* with or without NHP pretreatment.

Supplementary Figure 2 Representative images and lesion size measurements of wheat Bobwhite seedlings infected by *F. graminearum* with or without NHP pretreatment.

Supplementary Figure 3 The effects of NHP at indicated concentrations on *F. graminearum* growth in liquid medium.

Supplementary Figure 4 *F. graminearum* spores with propidium iodide staining for cell viability assessment.

Supplementary Table 1 Primers used in this study.

Supplementary Table 2 Detailed information of the upregulated wheat genes upon NHP treatment.

Supplementary Table 3 Expression data of the genes of interest.

Supplementary Table 4 Detailed information of GO terms that were overrepresented among the upregulated genes.

Supplementary Table 5 Detailed expression data of wheat genes encoding putative callose synthases.

Supplementary Table 6 Detailed information of GO terms that were overrepresented among the downregulated genes.
