# Supplementary material for: Transcriptomic Analysis of Wheat Seedling Responses to the Systemic Acquired Resistance Inducer N-Hydroxypipecolic Acid
Source: Front Microbiol. 2021 Feb 11;12:621336. doi: 10.3389/fmicb.2021.621336 (PMC7905219; doi:10.3389/fmicb.2021.621336)
Supplement: Supplementary Figure 1 — Representative images, lesion size measurements, and fungal abundance assessments of wheat cultivar Wangshuibai seedlings infected by F. graminearum with or without NHP pretreatment. [file Data_Sheet_1.PDF]

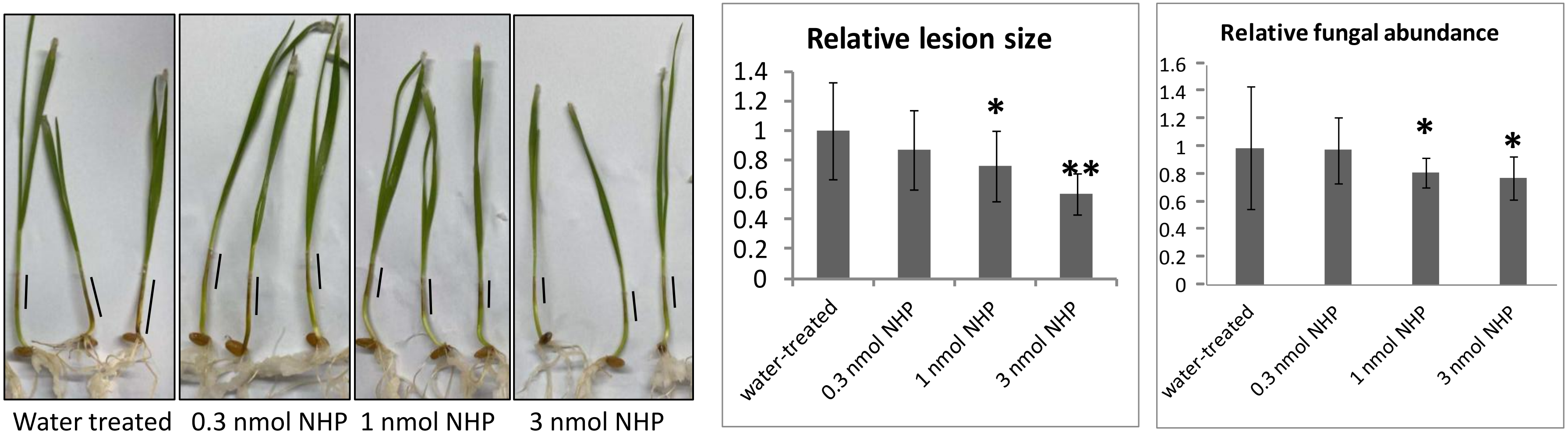

Supplementary figure S1. Representative images, lesion size measurements and fungal abundance assessments of wheat Wangshuibai seedlings infected by *F. graminearum* with or without NHP pretreatment. Lesions were indicated by black bars beside each seedling. Length of lesion of each seedling relative to average lesion size of water-treated (without NHP pretreatment) was measured. *, ** indicate significant difference between indicated sample and corresponding water-treated sample at P=0.05 and P=0.001 level, respectively (Student's *t*-test). Error bars represent standard deviation. Fourteen seedlings were measured in each treatment. Experiment was repeated twice, and results from one representative experiment were shown. Fungal abundance in infected tissue relative to water-treated sample were assessed by fungal genomic DNA PCR. Fourteen seedlings per treatment at 4 days after inoculation were pooled for genomic DNA extraction. Experiment was repeated twice, and results from one experiment were shown.

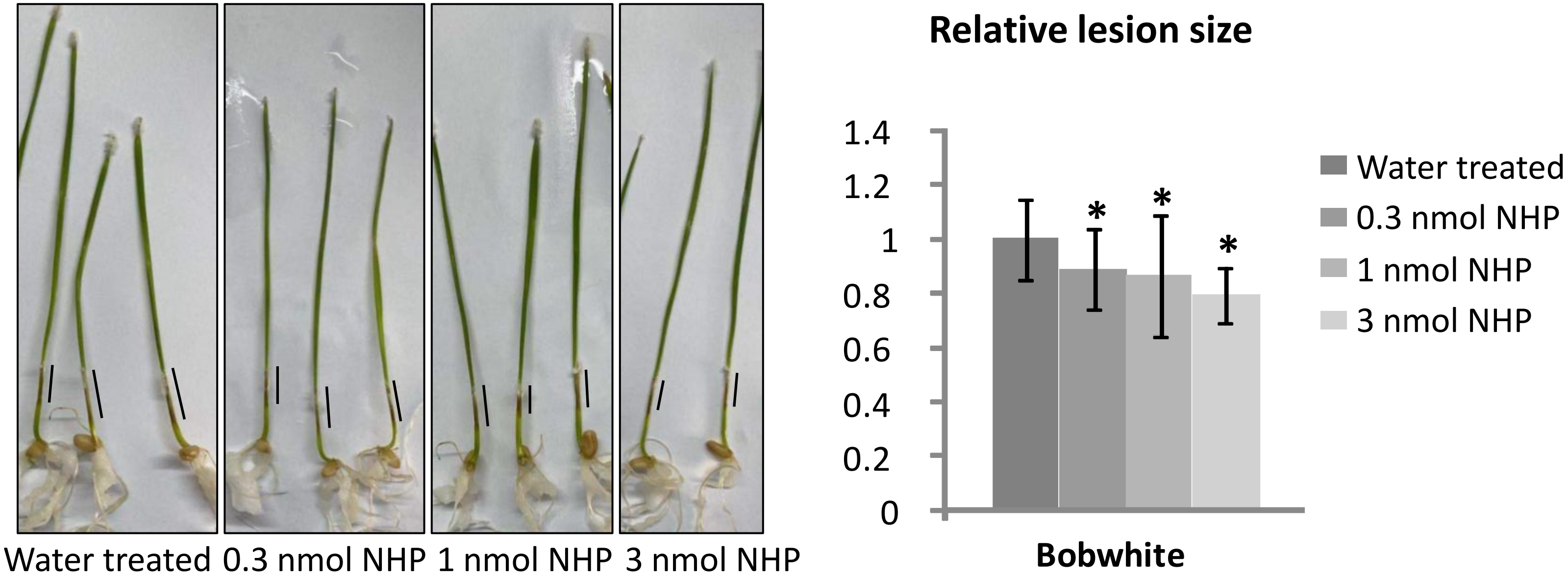

Supplementary figure S2. Representative images and lesion size measurements of wheat Bobwhite seedlings infected by *F. graminearum* with or without NHP pretreatment. Lesions were indicated by black bars beside each seedling. Length of lesion of each seedling relative to average lesion size of water-treated (without NHP pretreatment) was measured. *, significant difference between indicated sample and corresponding water-treated sample at P=0.05 level (Student t-test). Error bars represent standard deviation. Fourteen seedlings were measured in each treatment. Experiment was repeated twice, and results from one experiment were shown.

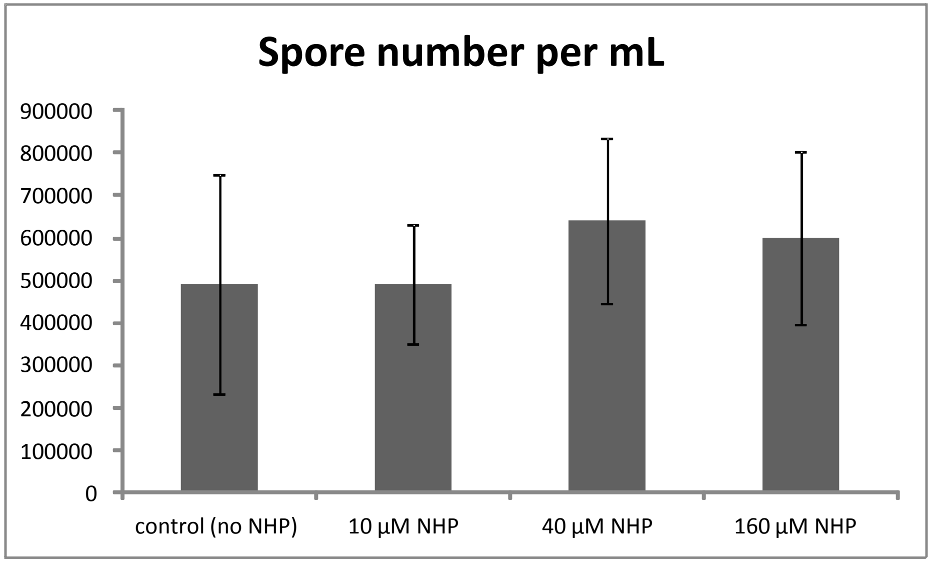

Supplementary figure S3. The effects of NHP at indicated concentrations on *F. graminearum* growth in liquid medium. Average spore numbers measured after 3 day culturing were charted . Error bars represent standard deviation. No significant difference were detected at P=0.05.

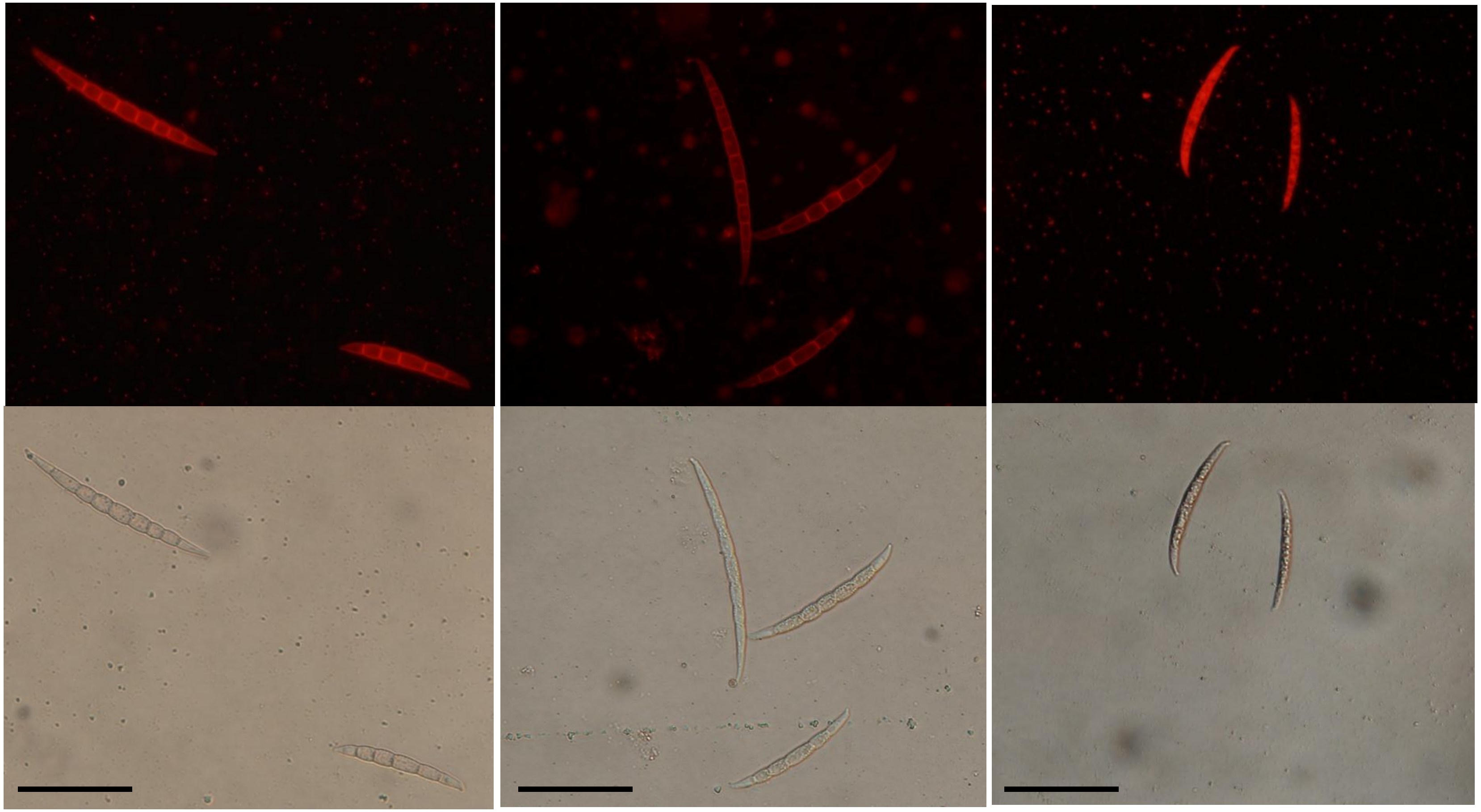

control (no NHP) 160 µM NHP Boiled dead spores

Supplementary figure S4. *F. graminearum* spores with propidium iodide staining for cell viability assessment. Spores cultured without (left) or with NHP (middle) were stained with 1.5 µM propidium iodide which emits red fluorescent light and can pass through disrupted membrane of dead cells. Right panel shows staining of dead spores. Scale bars = 50 µm.
